# BSR Annual Meeting 2018: Interventional Radiology

**DOI:** 10.5334/jbsr.1672

**Published:** 2018-11-17

**Authors:** Fabrice C. Deprez, Tom De Beule

**Affiliations:** 1 CHU UCL Namur, Godinne, BE; 2 AZ Sint Lucas, Gent, BE

**Figure:**
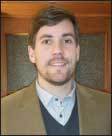
Fabrice Deprez, French representative (Chair) of the IR section of the BSR

**Figure:**
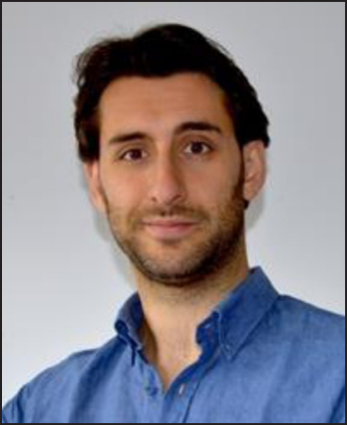
Tom de Beule, Flemish representative (Secretary) of the IR section of the BSR

Interventional radiology (IR) started to develop around the mid-1960s, when Charles Dotter, a pioneering U.S. vascular radiologist, performed the first angioplasty on a patient with gangrene and an ulcer on her foot. Since then, IR has expanded into almost all fields of medicine, “from head to toe”, concerning women, men, and children’s health at once. Today, IR is indisputably a medical sub-specialty, maintaining a strong growth potential, providing minimally invasive image-guided diagnosis and treatment by a wide range of techniques, constantly evolving.

IR is an essential part of radiology and constitutes a fantastic showcase of our specialty. Despite that, we still don’t have IR title recognition in Belgium, and consequently we don’t have a specific IR nomenclature, specific IR suites and equipment’s recognition, and a coherent identified nationwide IR offer. Moreover, with this situation, Belgian IR suffers from a fierce competition with other medical specialties in a very competitive and underfunded healthcare environment.

The IR section of the BSR is a very active and growing section of the BSR. One of our major goals is to improve IR networking, improve IR visibility and recognition regarding public and politics, and increase professional defense. For this reason, we endorsed and actively support the European Board of IR (EBIR) of the CIRSE (European Society of Cardiovascular and IR), and we encourage all our members, and especially the youngest, to obtain the EBIR certification.

In addition, we try to develop close relationships with the other European IR societies (especially with the French and the Dutch societies), which is why we are very proud to welcome very prestigious speakers at our BSR annual meeting.

As do all radiologists, interventional radiologists must be prepared to demonstrate their value to patients, providers, and the public. Our guests are all key opinion leaders in IR and will show us the best we can expect from IR!

Our first speaker, Professor Hicham Kobeiter, is the past president of the SFICV (Société Française d’Imagerie Cardiovasculaire et interventionnelle) and a worldwide-known expert in imaging guidance in IR. He is the Head of the department of radiology in the University Hospital Henri Mondor, in Créteil (France). His IR room is equipped with the latest technical developments of C-arm CBCT.

**Figure:**
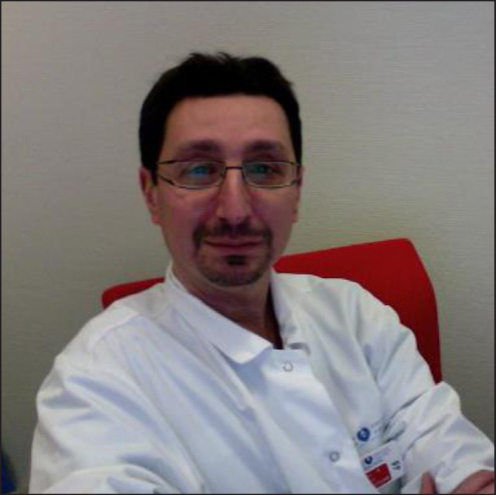
*Professor Hicham Kobeiter (MD, PhD) did his radiology residency in Paris (France), followed by a IR fellowship in Henri Mondor, Créteil (France), and a research fellowship in Johns Hopkins, in Baltimore (USA), with a special focus on IR. He is actually full Professor of Radiology and I, and Head of the department of Radiology in Henri Mondor.* *He is the past president of the SFICV and an active member of the CIRSE, ESCR (European Society of Cardiac Radiology), ESR (European Society of Radiology), and SFR (Société Française de Radiologie). He is consultant for the local and national committees of health “AFSAPPS, AGEPS and HAS (Haute Autorité de la Santé)”. He is a regular lecturer and teacher at international conferences and courses. He has published 118 peer-reviewed papers referenced on PubMed.*

Our second speaker, Professor Otto van Delden, is probably one of the most well-suited people in the world to talk about the role and the abilities of the Interventional Radiologist in 2018, as he is the Chairman of the European Board of IR (EBIR – CIRSE) and the chairman of the “Training & Education Committee” of the Dutch society of Radiology.

**Figure:**
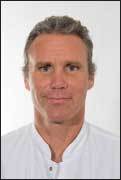
*Professor Otto van Delden (MD, PhD) did his radiology training at the Academic Medical Center (AMC) of the University of Amsterdam, followed by a fellowship in Vancouver, Canada. He has been an interventional radiologist since 1998 at the AMC, which is a tertiary referral center for cardiovascular disease and benign and malignant gastro-intestinal and hepatobiliary and pancreatic disease. He is currently focussing on training and education in IR, hepatobiliary interventions and interventional oncology.* *He was director of the AMC radiology residency program from 2005 to 2015, president of the Dutch society of interventional radiology (DSIR) from 2010 to 2017, a member of the scientific program committee of the cardiovascular and interventional radiological society of Europe (CIRSE) since 2010, the deputy-chair of the European board of interventional radiology (EBIR) of CIRSE from 2011 to 2015 and chair of EBIR since 2015, a member of the executive committee of CIRSE since 2015,and the chair of the training and education committee of the Dutch society of radiology since 2016.* *He has authored or co-authored more than 125 papers in peer-reviewed journals.*

Interventional radiology offers minimally invasive therapies that have clear advantages over surgical procedures. Radiological interventions are safer, better tolerated, and cheaper. One would assume that radiological interventions would be rapidly adopted once they have been shown to be equally effective as the surgical alternative. Not infrequently though, implementation of a radiological intervention into clinical practice is hampered, despite availability of scientific evidence demonstrating non-inferiority or even superiority over current standard of care.

Our final speaker, Professor Mark Burgman, is a very famous IR and the president of the Dutch Society of Radiology. He will discuss promising radiological interventions that could or should have been a treatment of first choice, but have thus far failed to have a significant impact on clinical management in the Benelux. The technique, indications and available scientific literature will be discussed of ‘misknown’ radiological interventions, such as percutaneous hepatic perfusion, renal tumor ablation, uterine artery embolization, thyroid ablation, and percutaneous placement of peritoneal dialysis catheters.

**Figure:**
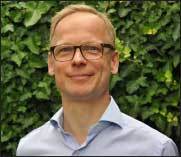
*Professor Mark Burgmans (MD, PhD) is a Dutch interventional radiologist at the Leiden University Medical Center and staff member at the Technical University Delft. His field of expertise is interventional oncology, in particular thermal tumor ablation, transarterial chemoembolisation, radioembolisation, and percutaneous hepatic perfusion. Professor Burgmans holds a PhD from the Leiden University (thesis ‘Advancements in minimally invasive image-guided liver therapies’) and is a principal investigator for studies on radiofrequency ablation with adjuvant Holmium radioembolisation, percutaneous hepatic perfusion with immunotherapy and quantitative ablation margin assessment using CT-CT co-registration. He is the current President of the Dutch Society of Radiology (DSR) and member of the board of the Dutch Hepatocellular and Cholangiocarcinoma Group (DHCG).*

